# Progress and Applications of Polyphosphate in Bone and Cartilage Regeneration

**DOI:** 10.1155/2019/5141204

**Published:** 2019-06-27

**Authors:** Yan Wang, Min Li, Pei Li, Haijun Teng, Dehong Fan, Wennan Du, Zhiliang Guo

**Affiliations:** ^1^The Second Department of Spine Surgery, The 80th Army Hospital of PLA (No. 89 Hospital of PLA), No. 256 Beigongxi Street, Weicheng District, Weifang 261000, Shandong, China; ^2^Department of Medical Affairs, The First Naval Hospital of Southern Theater Command of PLA, No. 40 Haibin 3rd Road, Xiashan District, Zhanjiang 524000, Guangdong, China; ^3^Department of Orthopedic Surgery, The 80th Army Hospital of PLA (No. 89 Hospital of PLA), No. 256 Beigongxi Street, Weicheng District, Weifang 261000, Shandong, China

## Abstract

Patients with bone and cartilage defects due to infection, tumors, and trauma are quite common. Repairing bone and cartilage defects is thus a major problem for clinicians. Autologous and artificial bone transplantations are associated with many challenges, such as limited materials and immune rejection. Bone and cartilage regeneration has become a popular research topic. Inorganic polyphosphate (polyP) is a widely occurring biopolymer with high-energy phosphoanhydride bonds that exists in organisms from bacteria to mammals. Much data indicate that polyP acts as a regulator of gene expression in bone and cartilage tissues and exerts morphogenetic effects on cells involved in bone and cartilage formation. Exposure of these cells to polyP leads to the increase of cytokines that promote the differentiation of mesenchymal stem cells into osteoblasts, accelerates the osteoblast mineralization process, and inhibits the differentiation of osteoclast precursors to functionally active osteoclasts. PolyP-based materials have been widely reported in in vivo and in vitro studies. This paper reviews the current cellular mechanisms and material applications of polyP in bone and cartilage regeneration.

## 1. Introduction

Inorganic polyphosphate (polyP) is a linear polymer of tens to hundreds of phosphate residues linked together via high-energy phosphoanhydride bonds. It exists widely in organisms from bacteria to mammals [1]. PolyP originally appeared during volcanic eruptions and was considered to be one of the first energetic molecules on Earth [2]. Initial researches showed that some microorganisms accumulated polyP in dense heterochromatic granules, which can be stained red with toluidine blue and be easily seen with an optical microscope [3]. As early as 1960, Fleish studied the role of polyP in tissue mineralization [4]. However, polyP was dormant for much of the 20th century [5], until Kornberg et al. developed a number of currently available methods for the detection and quantification of polyP [6, 7] and found the enzyme (polyphosphate kinase) for polyP synthesis in bacteria [8]. This major discovery of the kinase enabled them to manipulate polyP synthesis in organisms for the first time. It turns out that polyP has many functions in bacteria, such as maintaining bacterial survival, promoting energy metabolism, regulating gene expression [9], supporting translation fidelity [10, 11], and enhancing motility [12] and virulence [13]. In mammals, polyP is abundant in bodily fluids (synovial fluid and blood) and various cells (platelets, monocytes, fibroblasts, and osteoblasts) [14, 15]. This polymer also has a wide range of biological activities. It can act as a protein chaperone [16], regulate channel activity [17, 18], transmit metabolic energy [19], promote cell proliferation and differentiation [20], and ensure the stable expression of genes involved in differential gene expression [21]. Important functions related to bone and cartilage regeneration [22], coagulation [23, 24], and inflammation [25] have also been studied intensively. In the process of bone regeneration, polyP can promote osteoblast differentiation and calcification [26], inhibit the bone resorption activity of osteoclasts [27], and play a significant role in the positive regulation of bone tissue regeneration. This paper reviews the cellular mechanisms and material applications of polyP in bone and cartilage regeneration.

## 2. Chemical Structure and Characteristics of PolyP

PolyP is a linear polymer of tens to hundreds orthophosphate residues linked via phosphoanhydride P-O-P bonds [28, 29]. Microorganisms can produce polyP via polyphosphate kinases at ambient temperature [9], while the chemical synthesis of this polymer requires temperature of several hundred degrees [22]. The chain length of polyP varies considerably depending on the tissue and the organism in which it is produced [23, 29–32]. The mammalian brain tissue contains polyP of about 800 phosphates in length [29], while polyP within platelet-dense granules is narrowly distributed at about 60–100 phosphates long [29, 33]. When the chain length is less than 100 phosphate units, it readily dissolves in water at millimolar concentrations [9]. PolyP is stable over a wide pH and temperature range [9]; the degradation of polyP at physiological conditions and the absence of enzymes is pretty slow [34]. PolyP can be hydrolyzed by exophosphatase (PPX) and endophosphatase (PPN), which sequentially splits the terminal phosphates of polyP chain or cleaves internal phosphoanhydride bonds [23, 29, 30]. At physiological pH, each internal phosphate unit of polyP carries a monovalent negative charge, making polyP a strongly anionic polymer [32]. Furthermore, polyP may be one of the biopolymers containing the highest density of negative charge [28]. As a multivalent anion, polyP must form ionic bonds with both inorganic (Ca^2+^, Mg^2+^, Zn^2+^, Fe^2+^, Na^+^, etc.) and organic (basic amino acids, polyamines) cations [34]. It is a metal chelating agent [28].

## 3. PolyP and Mesenchymal Stem Cells

Mesenchymal stem cells (MSCs) are a kind of stem cells with a strong proliferative capacity and multidirectional differentiation potential. MSCs are mainly found in connective tissues and organ stroma and are most abundant in bone marrow tissues. They can differentiate into osteoblasts, chondrocytes, adipocytes, muscle cells, and other cells in a suitable in vivo or in vitro environment. Inorganic polyP has the ability to induce the differentiation of MSCs into osteoblasts [35].

Runt-related transcription factor 2 (Runx2) is the most critical transcription factor regulating MSC differentiation and maturation into osteoblasts during bone development and is regulated by bone morphogenetic protein 2 (BMP2) [36]. Runx2 is expressed in MSCs and during the different stages of the osteoblast lineage [37] and leads to stage-dependent increases in the expression of genes encoding a range of functional and structural proteins, such as alkaline phosphatase (ALP), collagen type I (COL-1), osteopontin (OPN), bone sialoprotein (BSP), osteocalcin (OC), and receptor activator of nuclear factor-*κ* B ligand (RANKL) [22]. Müller et al. [38] mixed a CaCl_2_ solution with a sodium polyP (Na-polyP) solution to precipitate amorphous calcium polyphosphate microparticles (Ca-polyP-MPs) with a diameter of 280±120 nm. They found that Ca-polyP-MPs promoted the growth of bone marrow-derived mesenchymal stem cells (BMSCs) and upregulated the expression of the transcription factors Runx2 and Sox9 (markers of chondrocyte differentiation) in bone marrow cells from rat femur explants. These transcription factors are critical for osteogenesis and chondrogenesis [39].

Fibroblast growth factor (FGF) plays a key role in bone regeneration [40]. Cell proliferation is one of the important steps in the early stage of bone formation. Kawazoe et al. [41] cultured human dental pulp cells (HDPCs) with pluripotent MSC characteristics and showed that polyP activated FGF-mediated cell signaling pathways by stabilizing FGF-2 and strengthening the affinity between FGF-2 and the receptors to promote the proliferation of HDPCs. PolyP not only physically and functionally stabilized FGF-2 but also prolonged the effect of FGF-2 on cells [41]. Microarray analysis showed that polyP induced the expression of matrix metalloproteinase-1 (MMP-1), OPN, OC, and osteoprotegerin (OPG) in HDPCs and human MSCs. Real-time PCR confirmed the induced expression of the MMP-1, OPN, and OC genes in both types of cells, and alizarin red S staining showed an enhanced degree of cell mineralization. Immunostaining analysis showed that polyP promoted the formation and maturation of COL-1. In short, polyP activates the FGF signaling pathway to induce the proliferation and osteogenic differentiation of stem cells [41].

Ozeki et al. [42] confirmed that polyP can induce the expression of osteoblast markers, such as ALP, OC, OPN, BSP, and osterix (OSX), in human adipose tissue-derived MSCs (hAT-MSCs), increase ALP activity and calcification capacity, and induce matrix metalloproteinase-13 (MMP-13) mRNA and protein expression. The transfection of an MMP-13 small interfering RNA (siRNA) effectively prevented the expression of the osteoblast markers ALP, OC, OPN, OSX, and BSP and blocked the osteogenic calcification of hAT-MSCs [42]. It was confirmed that polyP-induced MMP-13 can regulate the osteogenic differentiation of hAT-MSCs, which is essential for osteogenic differentiation. Similarly, the team found that matrix metalloproteinase-3 (MMP-3) has almost the same effect on rat dental pulp fibroblast-like cells (dental pulp stem cells). MMP-3 siRNA effectively inhibits the expression of osteogenic biomarkers, and polyP-induced MMP-3 regulates the differentiation of osteoblasts and rat dental pulp stem cells [43]. Further studies revealed that exogenous Wnt5 increased MMP-3 activity and accelerated the proliferation rate of dental pulp stem cells, while the transfection of a Wnt5*α* siRNA could inhibit polyP-induced MMP-3 expression and cell proliferation. This team demonstrated the sequential involvement of Wnt5 and MMP-3 in the polyP-induced proliferation of dental pulp stem cells, and MMP-3-mediated proliferation was mediated by the Wnt5 signaling cascade [44]. The Wnt family is highly conserved secretory glycoproteins that play important roles in regulating development, stem cell differentiation, proliferation, and apoptosis [45].

Wang et al. [46] found that polyP not only promoted osteogenesis but also promoted chondrogenesis. However, this activity was shown only in MSCs cultured with osteogenesis induction or cartilage induction. PolyP can upregulate the expression of the BMP-2, ALP, and COL-1 genes in MSCs after osteogenic induction and upregulate the expression of the collagen type II (COL-2) gene in MSCs after chondrogenic induction. However, MSCs cultured without osteogenic induction could not be stained by alizarin red S after adding polyP, and no cell mineralization was observed [46].

Platelet-rich plasma (PRP) promotes the expression of osteogenic markers (ALP and OC) [47] and chondrogenic markers (SOX9 and aggrecan) in MSCs [48, 49]. PRP also inhibits RANKL-induced osteoclast differentiation by activating the Wnt pathway during bone remodeling [50]. PRP contributes to the repair and regeneration of bone tissue, but its specific mechanism has not yet been fully elucidated. This effect is currently attributed to transforming growth factor-*β* (TGF-*β*), platelet-derived growth factor (PDGF), insulin-like growth factor (IGF), basic fibroblast growth factor (bFGF), vascular endothelial growth factor (VEGF), and other growth factors [48]. However, the content of polyP in platelet-dense particles is very high [15]. Since polyP is the main component found in platelets, it is reasonable to propose that this inorganic polymer plays a significant role in platelet-induced regeneration [32].

## 4. PolyP and Osteoblasts or Osteoblast-Like Cells

Osteoblasts are derived from MSCs, and hydroxyapatite (HA)-producing osteoblasts ultimately differentiate to osteocytes [51]. PolyP promotes the differentiation and calcification of osteoblasts [41, 52]. Müller et al. [53] cultured human osteosarcoma cells (SaOS-2) in a medium containing polyP and CaCl_2_ (2 mol: 1 mol). This polyP/Ca^2+^ complex could cause a strong increase in ALP activity and induce the steady-state gene expression of ALP (tissue nonspecific alkaline phosphatase (TNAP)). However, this effect was not observed in medium supplemented with inorganic phosphate alone. Immunocytological techniques found a large amount of ALP on the cell membrane, and membrane-bound ALP was localized in the matrix vesicles and the cell membrane, where HA crystallites grow [54]. Initially, HA crystallites were formed in the vesicles close to the cell membrane and eventually were released to the extracellular space [53]. The matrix vesicles contain polyP, and they are the initial sites of bone mineral formation [55]. Dexamethasone (DEX), ascorbic acid (AA), and *β*-glycerophosphate (*β*-GP) are essential substances in traditional osteogenic induction media. Müller et al. [53] found that *β*-GP can be replaced by a polyP/Ca^2+^ complex or inorganic phosphate. Even at a low concentration (100 *μ*M), the osteogenic induction effect of the polyP/Ca^2+^ complex was better than that of *β*-GP (10 mM), and the polyP/Ca^2+^ complex was superior to inorganic phosphate. This finding confirmed that it was not the organophosphate *β*-GP but the hydrolyzed inorganic phosphate (Pi) unit that served as a donor for HA formation. The authors reasonably hypothesized that polyP was hydrolyzed to Pi by the membrane-bound ALP outside the cell membrane and served as a substrate for HA crystallite formation. Omelon et al. [56] proposed a potential mechanism of mineralization regulation in which ALP cleaved polyP to Pi, making Pi available for HA formation. Bone cells biochemically control the production, placement, and activity of ALP and polyP to regulate HA mineralization. In an experiment, Müller et al. [53] found that the 100 *μ*M polyP/Ca^2+^ complex increased intracellular Ca^2+^ levels, whereas this effect was not found in the presence of polyP or inorganic phosphate alone. The authors believed that the ALP hydrolysis of the polyP/Ca^2+^ complex resulted in the release of both Pi and Ca^2+^ [53]. In response to the increase in extracellular Ca^2+^ concentrations, the intracellular Ca^2+^ level in osteoblasts increased [22]. The more important role of Ca^2+^ released during hydrolysis is to provide a calcium source for the formation of HA [54]. Usui et al. [57] also demonstrated that polyP can upregulate the expression of the genes encoding OC, OSX, BSP, and TNAP in mouse embryonic osteoblast precursor cells (MC-3T3-E1), induce osteoblast differentiation and calcification, and provide a material source for mineralization. In in vitro studies of the femur, Müller also found that polyP enhanced the mineralization of bone marrow cells in femoral explants [38].

Fibroblast growth factor 23 (FGF23) is mainly derived from mature osteoblasts and osteocytes [58]. It is a key regulator of mineral ion homeostasis [59] and plays an important role in inhibiting osteoblast mineralization [60]. Sun et al. [58] found that polyP can increase the phosphorylation of fibroblast growth factor receptor (FGFR), fibroblast growth factor receptor substrate 2 (FRS2), and extracellular regulated protein kinases 1 and 2 (Erk1/2). They demonstrated that polyP can upregulate the expression of the FGF23 gene and protein by activating the FGFR pathway. These results indicated that polyP may be related to mineral ion metabolism and that the increased expression of FGF23 may be the result of negative feedback regulation that inhibited mineralization induced by polyP.

Lui et al. [26] considered that extracellular polyP promoted proliferation, increased migration rate, prevented apoptosis, and significantly upregulated the expression of interleukin-11 (IL-11) at levels of mRNA and protein in SaOS-2 cells. PolyP directly stimulated the rapid phosphorylation of extracellular signal-regulated protein kinases (ERKs) via basic FGFR [26].

However, it has been found that *β*-GP and Pi can induce strong mineralization of SaOS-2 and MC-3T3 cells, while polyP, whether tested as units with a chain length of 17 or 42 phosphate units or as polyP or polyP/Ca^2+^ complexes, can induce cell mineralization [14]. Exogenous polyP did not enhance the deposition of mineral. Ariganello et al. [14] suggested that this process might involve cellular effects and physical interference rather than a lack of active ALP, and unless polyP can be processed, its mineral inhibitory capacity was dominant.

## 5. PolyP and Osteoclasts or Osteoclast-Like Cells

There are a large number of particles that contain polyP in osteoclasts [61]. Osteoclasts are derived from hematopoietic stem cells. Their main stages of differentiation include preosteoclasts and mature osteoclasts with bone resorption activity [22]. To scavenge and store the free orthophosphate released during the process of bone mineral resorption, osteoclasts can store high concentrations of orthophosphate in the mitochondrion by condensing orthophosphate ions into polyP [1].

The marker of terminally differentiated mature osteoclasts is tartrate-resistant acid phosphatase (TRAP) [22]. The bone resorption activity of osteoclasts is dependent on TRAP, which is encoded by mammalian Acp5 gene and translated into a 35 kDa monomeric enzyme [62]. Subsequently, this monomeric enzyme is hydrolyzed into 22 kDa N-terminal and 16 kDa C-terminal fragments, which are then disulfide-bonded to form an active heterodimeric enzyme [63]. During bone resorption, TRAP is released from the basolateral surface into the extracellular space and enters the resorption lacuna from the ruffled border [27]. TRAP can dephosphorylate many substrates, including OPN, BSP, casein (CAS), and mannose 6-phosphate (M6P), but the specificity of TRAP substrates is not high. TRAP also has polyphosphatase activity and can degrade polyP. In addition, short-chain polyP has better degradation efficiency than long-chain polyP [27]. Due to competitive inhibition of the enzyme, polyP inhibits the phosphatase activity of TRAP in osteoclasts and inhibits the bone resorption activity of osteoclasts. In addition, long-chain polyP (polyP300, 300 phosphate residues) can bind TRAP with a higher affinity than short-chain polyP (polyP15, 15 phosphate residues), and its inhibitory effect is significantly stronger than that of short-chain polyP. In contrast, osteoclasts can also degrade polyP, creating favorable conditions for bone resorption [27]. In terms of osteoclast differentiation, Harada [27] noted that polyP had no inhibitory effect. Conversely, long-chain polyP (polyP300) promoted osteoclast precursor division and enhanced the number of TRAP-positive (TRAP+) multinucleate cells.

(Pre)osteoclasts express the receptor activator of NF-*κ*B (RANK) in the presence of dihydroxyvitamin D3. The interaction between RANKL produced by osteoblasts and its receptor RANK can induce (pre)osteoclasts differentiation [22, 64]. In addition, the osteoclasts become functionally active, and the degradation of HA occurs after RANKL binds to RANK. This process is essential for osteoclastogenesis and the activation of osteoclasts [22]. I*κ*B*α* kinase is a key molecule mediating the activation of NF-*κ*B during RANKL-induced (pre)osteoclast differentiation [65]. Immunoblotting studies revealed extensive phosphorylation of I*κ*B*α* on osteoclast-like RAW 264.7 cells that had been incubated in the presence of RANKL but the absence of the polyP/Ca^2+^ complex. However, the addition of the polyP/Ca^2+^ complex strongly inhibited the phosphorylation of I*κ*B*α* kinase, even at a low concentrations of 10-100 *μ*M [66]. The phosphorylation of I*κ*B*α* kinase is needed for NF-*κ*B activation [65, 67]. It was concluded that the polyP/Ca^2+^ complex interfered with RANKL-mediated NF-*κ*B activation at the I*κ*B*α* kinase level and impaired the differentiation of preosteoclasts into functional osteoclasts [66]. In addition, the polyP/Ca^2+^ complex can reduce the number of TRAP+ cells at concentrations >10 *μ*m [66], which is contrary to the viewpoint of Harada et al. [27].

The expression level of the cathepsin K gene is considered to be a reliable marker of osteoclast function. Müller et al. [38] found that Ca-polyP-MPs and Ca-polyP/zoledronic acid (Zol) hybrid microparticles (Ca-polyP-Zol-MPs) can downregulate the expression of cathepsin K in osteoclasts after 7 days of incubation. However, the MPs did not affect the expression of TRAP.

## 6. PolyP and Cartilage or Chondrocytes

Articular cartilage is a connective tissue that covers the surfaces of the synovial joints. Due to its avascular nature, it is difficult to repair itself after injury [68]. St-Pierre et al. [69] demonstrated that polyP increases the expressions of glycosaminoglycan (GAG) and COL in a chain length- and concentration-dependent manner in both cell cultures and cartilage tissue cultured ex vivo. The effect of 1 mM polyP was superior to that of 0.1 mM polyP, 0.5 mM polyP, and the untreated control condition. Additionally, polyP with an average chain length of 45 phosphate units significantly increased GAG and COL contents compared to those of cultures exposed to 5 phosphate units, 75 phosphate units, and the untreated control condition. While the mechanism by which polyP promotes the deposition of cartilage matrix has not yet been determined, the authors proposed that polyP may act by stabilizing endogenous growth factors [69]. However, treatment of ex vivo-formed cartilage with polyP did not upregulate the expression of cartilage matrix genes aggrecan and COL-2. The authors thus suggested that polyP inhibited chondrocyte proliferation [69]. However, polyP has been shown to stimulate the proliferation of HDPCs and human fibroblasts through the FGF signaling pathway [41, 70]. This discrepancy might be due to cell-type-specific effects [69].

When cultured on Ca-polyP, a porous material, chondrocytes maintain a polygonal shape and show a ring-like distribution of actin filaments; in contrast, chondrocytes are flattened and exhibit actin filaments dispersed throughout the cytoplasm when cultured on polystyrene [71]. Ciolfi et al. [71] suggested that Ca-polyP may limit cell spreading by maintaining a ring-like actin distribution.

## 7. PolyP in Cell Metabolism

Glycolysis is the main pathway through which cells produce metabolic energy in the form of ATP during bone formation. Lactic acid is the end product of glycolysis under hypoxia, and its content reflects the metabolic level. Carbonic anhydrase (CA) catalyzes the reversible conversion of CO_2_ to HCO_3_^−^ as an internal buffer to regulate and control pH [72]. Müller et al. [72] found that the activity of SaOS-2 cells and the production of lactic acid were significantly enhanced with increasing polyP concentrations (0 *μ*g/ml to 30 *μ*g/ml) under hypoxic culture conditions. The gene expressions of carbonic anhydrase IX (CA IX) and hypoxia inducible factor 1*α* (HIF-1*α*) were significantly upregulated at the mRNA level after 3 days of culture with 10 *μ*g/ml polyP under hypoxic (8% oxygen tension) conditions. The transcriptional proteins (CA IX and HIF-1*α*) also increased after 5 days of culture with 10 *μ*g/ml polyP under hypoxic conditions. Finally, the authors confirmed that even under hypoxic conditions, the calcification of SaOS-2 cells could reach an aerobic level after adding polyP, which is necessary for bone mineralization under physiological hypoxic conditions [72].

PolyP is an energy-rich linear polymer with tens to hundreds of phosphoric anhydride bonds. Cells can absorb polyP by endocytosis [73]. Müller et al. [74] found that SaOS-2 cells can produce a large amount of ATP in a polyP-rich environment. After osteogenic induction and incubation with polyP, the intracellular ATP level can be increased by 10-fold. The ATP can be strongly released into the extracellular space, resulting in an increase in extracellular ATP levels. It was confirmed that the energy released by the hydrolysis of high-energy phosphoric anhydride bonds can be utilized by cells as metabolic fuel to facilitate the formation of HA on the extracellular plasma membrane [74]. Further studies revealed that ALP and adenylate kinase (AK) were released into the extracellular space through the transport of matrix vesicles and translocated to the cytomembrane in response to polyP. The authors believed that the high-energy phosphoric anhydride bonds of polyP were hydrolyzed by ALP, releasing metabolic energy to form ADP, which was then used by AK to synthesize ATP, as ALP and AK are involved in the synthesis of extracellular ATP and ADP [75]. PolyP releases the Gibbs free energy through ALP-mediated enzymatic hydrolysis to form ATP both inside and outside the cell. Therefore, polyP can be considered an energy reservoir [38] and can be used as an alternate energy source for some organisms that are exposed to anoxic environments [1].

## 8. Application of PolyP Materials

### 8.1. Metal Cationic Polymers

As a multivalent anion, polyP must bind to the metal cations. This polymer can act on cells in two forms: soluble salt solutions (e.g., sodium polyphosphate, Na-polyP) and granular nanoparticles (e.g., Ca-polyP-MPs). Moreover, polyP can be used as a strong chelator of Ca^2+^ ions [9]. Wang et al. mixed Na-polyP with CaCl_2_ (2 mol: 1 mol) to form a polyP/Ca^2+^ complex to compensate for the effect of the resulting chelation on Ca^2+^ ions after polyP was potentially hydrolyzed into monomeric phosphate or pyrophosphate by phosphatase in vitro [53, 66]. This chelating effect can reduce the concentration of free Ca^2+^ ions in culture and may even deplete the Ca^2+^ required for HA deposition [14]. It was found that this polyP/Ca^2+^ complex was more conducive to the mineralization of SaOS-2 cells and the expression of BMP-2 than Na-polyP [66].

Müller et al. dissolved 2.8 g of CaCl_2_·2H_2_O and 1 g of Na-polyP in 25 ml of distilled water and then added the CaCl_2_ solution dropwise into the Na-polyP solution. The pH of the suspension was adjusted to 10. Finally, the solution was stirred, filtered, washed with ethanol, and dried, and Ca-polyP-MP was obtained [38, 72, 76]. Compared with Na-polyP, which is dissoluble at physiological pH, Ca-polyP becomes insoluble and gradually hardens with increasing concentrations and chain lengths, This is a prerequisite for a material used in bone tissue engineering [22]. At present, Ca-polyP is the most widely used polyP in bone regeneration research [38, 72, 74]. As a bioactive ceramic, its elemental composition is similar to that of inorganic matter in natural bone tissue. In addition, Ca-polyP has good bioactivity, biocompatibility, degradability, and mechanical properties [77].

It has been shown that strontium ions can stimulate osteoblast activity, promote bone formation, and inhibit bone resorption [78, 79]. Müller et al. [80] dissolved 1 g of Na-polyP in 50 ml of distilled water and dissolved 5.16 g of SrCl_2_·6H_2_O in 50 ml of ethanol. Then, they mixed Na-polyP solution and strontium solution, adjusting PH to 10. Finally the solution was precipitated, filtered, washed, and dried to obtain amorphous Sr-polyP-microparticles (Sr-polyP-MPs). The bone regeneration effect of Sr-polyP-MPs was studied in vivo and in vitro. The results showed that Sr-polyP was superior to Ca-polyP in promoting MSC growth/metabolic activity, inducing the mineralization of SaOS-2 cells, and enhancing the expression of the ALP and BMP-2 genes in vitro. Moreover, compared with Sr-polyP, Ca-polyP significantly upregulated the expression of osteocyte-specific sclerostin (a negative regulatory factor of the Wnt signaling pathway and an inhibitor of the differentiation and mineralization of bone cells) [80]. In an in vivo study, poly(d,l-lactide-co-glycolide) (PLGA) particles containing polyP microspheres were implanted into critical-size calvarial defects of rats. The results showed that the osteogenic effect of Sr-polyP was also superior to that of Ca-polyP [80].

Wang et al. [81] prepared Gd-polyP by mixing Na-polyP with GdCl_3_ at a molar ratio of 3:1. After incubating the SaOS-2 cells with Gd-polyP, they found that 5 mM Gd-polyP significantly promoted the mineralization of SaOS-2 cells, enhanced ALP activity, and upregulated the expression levels of the BMP-2 and COL-1 genes compared with the control groups, 5 mM Ca-polyP and 5 mM GdCl_3_. Thus, Gd-polyP has good osteogenesis-induced morphogenesis activity [81].

### 8.2. PolyP Hybrid Composites

Li et al. [77] doped copper (Cu) into Ca-polyP and used the formed Ca-polyP/Cu hybrid scaffolds to conduct experiments on bone defect repair. These scaffolds that incorporated Cu showed improved mechanical strength, cytocompatibility, and proliferation activity of cells. However, the incorporated Cu failed to change the degradation velocity of the scaffold. After Cu was doped, the expression of HIF-1*α*, ALP, OC, and VEGF was significantly upregulated both in vitro and in vivo. The combination of the Ca-polyP/Cu hybrid composite and preconditioned BMSCs further enhanced new bone formation. In conclusion, doping Cu into Ca-polyP was a new strategy for improving the mechanical properties of Ca-polyP and promoting its osteogenesis and angiogenesis potential. Ma et al. [82] assessed the degradation, biocompatibility, and osteogenesis of the lithium (Li) doped Ca-polyP scaffolds. They found that the hybrid scaffolds had better biodegradability and compressive strength, and 2% Li-doped Ca-polyP was most beneficial to cell growth and attachment.

Qiu mixed [83] strontium into Ca-polyP. It was confirmed that the doped strontium did not change the structure of Ca-polyP. This hybrid polymer can promote the proliferation and growth of osteoblasts in vitro. In in vivo studies, compared with Ca-polyP scaffolds, this hybrid polymer scaffold can increase the expression of COL-1 and BMP and has similar degradability [84]. Osteogenesis is closely related to VEGF and bFGF. Liu et al. [85] cultured osteoblasts with Ca-polyP doped with different doses of strontium and found that the mRNA expression and protein levels of VEGF and bFGF in cultured osteoblasts increased in a dose-dependent manner until the optimal dose (8% mol) was reached. It has been shown that Sr-doped Ca-polyP can induce angiogenesis and can be used as a good biomaterial in the context of bone tissue engineering and bone repair [85]. Qin et al. [86] also confirmed that Ca-polyP scaffolds containing 1% mol of strontium were more favorable to the proliferation and growth of HDPCs than pure Ca-polyP and HA scaffolds. ELISA analysis revealed that the protein levels of VEGF and bFGF in HDPCs cultured on these scaffolds were significantly higher than those in HDPCs cultured on Ca-polyP and HA scaffolds. A 1% Sr-doped Ca-polyP scaffold may induce angiogenesis and have better cytocompatibility in HDPCs [86]. Gu et al. [87] further confirmed that porous Sr-doped Ca-polyP scaffolds could accelerate bone formation by stimulating human osteoblast-like cells (MG63) to secrete VEGF and bFGF in in vitro and in vivo studies. When combined with MSCs, these scaffolds could further accelerate the repair of rabbit segmental bony defects. The release of strontium ions may be the reason why this hybrid polymer has better osteogenic capacity than pure Ca-polyP [84]. Similarly, compared with Sr-doped Ca-polyP, the implantation of a combination of Sr-doped Ca-polyP and autologous bone marrow mononuclear cells can significantly enhance VEGF expression and promote osteogenesis, but the effect was not as good as that of morselized autogenous cancellous compact bone grafts [88].

There is a large amount of HA in the bones of vertebrates. Natural or synthetic HA is widely used as a scaffold material for bone tissue and other tissue engineering purposes due to its biocompatibility [89]. Müller et al. [89] added different concentrations of Na-polyP during the synthesis of HA. It was found that the crystallization of HA could be inhibited, and polyP/HA hybrid particles with a size of approximately 50 nm could be formed at higher concentrations of polyP (mass > 10%). Compared with HA, the polyP/HA hybrid particles, which had bioactivity similar to that of Ca-polyP nanoparticles, could enhance the metabolic activity of SaOS-2 cells and human MSCs and upregulate the gene expression of COL-1 and ALP, providing a better design and improved selection for bone tissue engineering materials. PolyP adsorbed on the surface of an HA plate can also enhance the calcification of the cultured osteoblasts [90]. Wang et al. [91] confirmed that the mixing of amorphous calcium carbonate particles with amorphous Ca-polyP particles can enhance the proliferation rate of human MSCs in in vitro studies. An in vivo model study of rat critical-size calvarial defects confirmed that the mixed particles of these two amorphous substances can accelerate the bone mineralization process and upregulate the expression of marker genes (compared to Ca-polyP particles alone). These results indicated that calcium carbonate particles can enhance the capacity of polyP to induce bone regeneration in vivo, which reveals a new strategy for the development of regenerative implants for the reconstruction of bone defects.

Dhivya et al. [20] studied the proliferation and differentiation of BMSCs on a bone tissue engineering scaffold composed of chitosan (CS), Ca-polyP, and pigeonite (Pg). They found that BMSCs had stronger proliferation activity on the CS/Ca-polyP/Pg scaffold than on the CS/Ca-polyP scaffold or in the blank control group. This tissue engineering scaffold promoted osteoblast differentiation at the cellular and molecular levels, enhanced calcium deposits, and increased osteogenic marker gene expression (Runx2, ALP, COL-1, and OC) and ALP activity. The authors implanted the scaffold into an in vivo model of rat 3 mm critical-sized tibial defects. The bone formation of the rats was significantly enhanced in scanning electron microscopy (SEM), energy dispersive X-ray analysis (EDAX), and X-ray radiographs analyses after 8 weeks [20]. Luo et al. [92] developed a new Ca-polyP composite scaffold that contained Li and VEGF-loaded gelatin microspheres. MSCs were seeded on scaffolds in vitro. The results showed that this composite scaffold had better efforts of osteogenesis and angiogenesis. In a rabbit model of glucocorticoids-induced osteonecrosis of femoral head, radiographic, histological analysis and western blot analysis showed that this composite scaffold also had better efforts of osteogenesis and angiogenesis than blank control group, autologous bone group, and Li-Ca-polyP group [92].

Zol can promote apoptosis in cancer stem cells (CSCs) by upregulating proapoptotic genes and downregulating antiapoptotic genes [93]. This approach has the effect of inhibiting tumor bone metastasis [38, 94]. Müller et al. [38] found that the anionic drug Zol can be embedded in Ca-polyP-MPs by Ca^2+^ ion bonds to form diamond-shaped Ca-polyP-Zol-MPs. PolyP reversibly binds to drugs and participates in drug delivery systems to treat bone metastases. This hybrid particle not only exhibited the cytotoxicity of bisphosphonate, but also showed mineralization-inducing and morphogenetic activity of polyP, which was suitable for targeted application in bone tumors and their metastases. Wu et al. [95] conjugated polyP onto hyaluronic acid macromers, developing a novel bioactive hydrogel that can provide constant osteoconductive stimulation to MC-3T3-E1 cells; this hydrogel has a higher osteoconductive activity than the free-floating polyP, resulting in the upregulation of osteogenic marker genes and the enhancement of ALP activity. At pH 7.4, the Ca-polyP nanoparticles had a high zeta potential of -34 mV, which contributed to the stability but impaired the biocompatibility of the nanoparticles (radius 94 nm) suspensions [96]. After Müller et al. [96] exposed Ca-polyP nanoparticles to serum containing protein/peptides, the zeta potential could be reduced to near zero, and the particles quickly transitioned to a coacervate phase. The cultured MSCs could be embedded in the coacervate and had enhanced growth/proliferation activity.

In terms of 3D printing, Ca-polyP and poly-*ε*-caprolactone (PCL) can be mixed to form 3D printing biomaterials. The printed hybrid scaffold has not only biomechanical properties but also morphogenetic activity, which can promote the growth and mineralization of SaOS-2 cells [97]. Wang et al. [46] found that polyP was a morphogenetically active additive for the bioinert alginate polymer. Alginate supplemented with polyP was a suitable material that enhanced the proliferation and differentiation of human MSCs. This biomaterial has potential application value in 3D tissue printing. This research team further found that the addition of the polyP/Ca^2+^ complex and agarose to the alginate/gelatin hydrogel caused an obvious enhancement in the hardness and calcification of the cells and could be used as biomaterial for 3D tissue printing, which opens up a new possibility for the application of 3D bioprinting in bone tissue engineering [98].

Müller et al. [99] made a bioinspired hydrogel material with morphogenetic activity and viscoelastic properties matching those of cartilage with Na-polyP, N,O-carboxymethyl chitosan, and alginate. This hydrogel material upregulated the expression of the genes encoding COL-2, ALP, and aggrecan in SaOS-2 cells and can be used to make 3D solid models of cartilage. In another study, Müller et al. [100] dissolved 1 g of hyaluronic acid, 1 g of Na-polyP, and 0.5 g of CaCO_3_ in 10 ml of distilled water. Subsequently, 5 ml of distilled water containing 1.5 g of solid MgCl_2_•6H_2_O was added. As a result, the authors obtained a fluffy and microporous hyaluronic acid-Mg/Ca-polyP paste. This material can bind Ca^2+^ in the synovial fluid, upregulate the expression of the genes encoding ALP, COL-2*α*1, COL-3*α*1, and aggrecan in human chondrocytes, and settle chondrocytes and stimulate them to reach a higher proliferative state. This material can also be used as an artificial cartilage material with regenerative activity [100]. Wang et al. [76] added Mg^2+^ ions to a solution that contained hyaluronic acid and soluble Na-polyP and formed a three-component system (HA-Mg^2+^-Na-polyP) that was highly viscous with a paste-like consistency. This matrix material strongly promoted the adhesion of chondrocytes to the culture dishes, even in comparison to HA alone. Compared with those of the blank control, Na-polyP, and HA groups, the transcript levels of SOX9 and COL3A1 were significantly upregulated in chondrocytes during a 21-d incubation period with this material. When chondrocytes were cultured in synovial fluid containing polyP with about 80 phosphoric acid residues, the expression of COL3A1 gene was also significantly increased. However, this stimulation was eliminated after the synovial fluid was incubated with polyP-degrading ALP. These observations provided strong experimental evidence that polyP is a key regulator of the metabolic activity of chondrocytes.

Articular cartilage interfaces with bone through a zone of mineralized cartilage that mediates the mechanical force transfer from soft cartilage to hard bone to better resist interfacial shear forces [101]. To simulate such zonal organization, some researchers devised a biphasic construct consisting of chondrocytes from articular cartilage anchored to the surface of porous Ca-polyP [102–105]. After 8 weeks of incubation, the cartilage layer formed on the Ca-polyP surface and within the pores near the surface and was mechanically anchored to the Ca-polyP [103]. Thus, a mineralized cartilage zone was generated within the Ca-polyP-cartilage interface [104]. It was found that this biphasic construct formed has better interfacial shear strength and cartilage load-bearing capacity [102] and can enhance the mechanical integrity of the cartilage-bone interface [104]. Such biphasic constructs could successfully repair focal subchondral defects in sheep joints [103]. PolyP released from Ca-polyP inhibits the calcification of cartilage in a chain length- and concentration-dependent manner [104]. Depositing a calcium phosphate film on Ca-polyP by a sol-gel process can prevent the accumulation of polyP and the associated inhibition of mineralization [106]. Lee et al. [101] prepared an “osteochondral-like” implant with cartilage cells and hydroxyapatite-coated Ca-polyP to simulate this zonal organization. The production of a layer of mineralized cartilage at the interface could help this “osteochondral-like” implant to repair cartilage defects [101].

## 9. PolyP and Other Biomaterials

Biomimetics, biocompatibility, biodegradability, and mechanical properties should be considered in the design of scaffolds for bone tissue engineering [107]. There are several types of biomaterials currently in use or in development for bone tissue engineering. (1) Naturally derived biomaterials (collagen, chitosan, hyaluronic acid, alginate, and fibrin, etc.) are produced by living organisms. These biomaterials are inherently biocompatible, showing minimal adverse immunogenicity, and are readily available from natural resources [108, 109]. However, they usually lack the required mechanical strength [107]. (2) Synthetic biomaterials [polylactic acid (PLA), polyglycolic acid (PGA), and their copolymers poly(DL-lactic acid-co-glycolic acid, PLGA), poly-*ε*-caprolactone (PCL), etc.] can be large-scale produced, be designed to precise geometric forms, and show desired and predictable mechanical properties and biodegradability [107]. However, they are limited in their applicability to bone regeneration due to their low osteoinductive capacity [107] and higher incidence of inflammatory reactions [110]. (3) Ceramics [hydroxyapatite (HA), calcium sulfate (CS), calcium carbonate, dicalcium phosphate, octacalcium phosphate, etc.] consist of inorganic oxides and salts. In contrast, bioceramics have good osteoconductivity, high compressive strength, and good osseointegration [111, 112]. However, this material has low osteoinductive activity, high brittleness, weak shaping ability, and long degradation time [113]. PolyP is widespread and produced in organisms. It can also be synthesized by chemical methods and has properties of bioceramics. In a word, polyP has the advantages of the above three biomaterials in osteogenesis. It has been proved that the composites of polyP and other biomaterials had better mechanical properties and biological functions than polyP or other biomaterials alone [76, 89, 91].

## 10. Summary and Prospects

PolyP is a biopolymer that is found in almost all cells and tissues. Because of its morphogenetic activity and role as a metabolic fuel inside and outside of cells, the application of polyP has become a key topic in current research. In the field of new biomaterial research on bone and cartilage repair, polyP-based materials have broad application prospects [15]. However, the specific biological functions of polyP have not yet been fully elucidated in many fields, including bone and cartilage regeneration [27, 114]. The process and specific enzymatic mechanism by which polyP is synthesized and consumed during bone and cartilage regeneration require further study. In addition, how polyP is transported and redistributed in cells and organelles remains unknown. The detailed mechanism of each stage of bone regeneration requires further clarification. There is an urgent need to further explore how to optimize the use of polyP and its composites in bone and cartilage tissue engineering. In summary, this paper reviews the current cellular mechanisms and material applications of polyP in bone and cartilage regeneration. PolyP is expected to play an important role in bone and cartilage tissue engineering in the future.
